# Increased dairy product consumption is associated with shorter telomere length in buccal cells among normotensive adults

**DOI:** 10.37796/2211-8039.1692

**Published:** 2026-03-01

**Authors:** Hsin-Hwa Tsai, Yang-Di Su, Zi-Lun Lai, Chia-Yu Lin, Shu-Fan Lin, Hsiu-Ching Hsu, Wen-Yuan Lin, Po-Ren Hsueh

**Affiliations:** a Department of Laboratory Medicine, China Medical University Hospital, China Medical University, Taichung, Taiwan; b Department of Family Medicine, China Medical University Hospital, China Medical University, Taichung, Taiwan; c Department of Internal Medicine, China Medical University Hospital, China Medical University, Taichung, Taiwan

**Keywords:** Aging, Telomere length, Dairy consumption, Hypertension, Cross-sectional study

## Abstract

**Background:**

Telomere length (TL) is a biomarker of biological aging and a predictor of age-related diseases. Dietary patterns, including dairy consumption, may influence telomere dynamics, but the evidence remains limited, particularly in Asian populations. This study investigates the association between dairy consumption and relative telomere length (RTL) in Taiwanese adults, with results analyzed by hypertension status.

**Methods:**

A cross-sectional survey was conducted among 259 adults in Taipei, Taiwan. RTL was measured using quantitative PCR from buccal cells. Dietary intake was assessed through self-reported questionnaires, with a focus on dairy frequency and fat content. Multiple linear regression models were used to examine the association between dairy intake and TL, adjusting for demographic and lifestyle factors.

**Results:**

Among normotensive individuals, higher frequency of dairy consumption was significantly associated with shorter RTL (β = −0.082, *p* < 0.01), particularly with low-fat and fat-free dairy products (β = −0.106, *p* < 0.01). No significant associations were observed in the hypertensive group.

**Conclusion:**

Dairy intake, particularly of low-fat products, may contribute to telomere shortening in normotensive adults. This association was not evident in hypertensive individuals, possibly due to a ceiling effect of chronic inflammation. These findings highlight the need for individualized nutritional guidance in public health strategies targeting healthy aging.

## 1. Introduction

Telomeres are nucleoprotein complexes that cap the ends of chromosomes and preserve genomic stability by preventing DNA degradation, end-to-end fusions, and inappropriate activation of DNA damage responses [1]. In mammals, telomeres consist of repetitive (TTAGGG) sequences bound by shelterin proteins. Telomere length (TL) progressively shortens with each somatic cell division, primarily due to the end-replication problem and limited telomerase activity. TL attrition is widely recognized as a reliable biomarker. Notably, telomere shortening is not solely a consequence of replication but is also driven by external and intrinsic stressors such as oxidative damage, chronic inflammation, and replication stress [2]. Shortened telomeres have been associated with an elevated risk of age-related diseases such as cardiovascular disease, neurodegenerative disorders, and various cancers [3]. These associations highlight the central role of telomere dynamics in both aging processes and disease development. Although TL varies by tissue type and individual characteristics due to genetic and environmental factors, the general trend is a gradual decline in TL with age. Understanding the molecular mechanisms that regulate telomere maintenance and attrition is therefore essential for identifying potential targets for interventions aimed at slowing age-related physiological decline.

Hypertension is one of the most prevalent chronic conditions in older adults and a leading contributor to global mortality, affecting approximately 30 % of the adult population [4]. As a major risk factor for cardiovascular, cerebrovascular, peripheral vascular, and renal diseases, hypertension is strongly associated with diminished quality of life, particularly among older adults. Increasing evidence indicates that telomere dysfunction, especially the age-associated shortening of leukocyte telomere length (LTL), may be involved in the pathophysiology of hypertension [5–7]. Individuals with essential hypertension (EH) have been found to exhibit significantly shorter LTL compared to normotensive individuals, suggesting a molecular connection between cellular aging and elevated blood pressure [8]. Oxidative stress and chronic inflammation, which are commonly observed in hypertensive individuals, have been proposed as potential biological mechanisms linking telomere shortening with hypertension. Telomeres are particularly susceptible to oxidative DNA damage, and sustained exposure to physiological stress may accelerate their attrition [9]. Although oxidative injury is known to compromise telomere maintenance and DNA repair processes, the specific biological pathways that link telomere shortening with hypertension and other systemic outcomes remain incompletely understood.

Among dietary factors, dairy consumption, particularly milk, has gained attention for its complex and often contradictory health implications. Dairy products are important sources of nutrients such as calcium, protein, and vitamins, and it is often promoted as part of a healthy diet. Several epidemiological studies suggest that moderate consumption of milk or dairy products may be associated with lower risks of cardiovascular disease, stroke, hypertension, metabolic syndrome, osteoporosis, and Alzheimer’s disease [10]. Some findings also indicate that moderate intake of milk fat may improve cardiovascular profiles by modifying low-density lipoprotein (LDL) particle size [11]. However, other studies raise concerns about potential adverse effects of dairy, suggesting that certain components such as saturated fat and galactose may contribute to inflammation, metabolic disturbances, and oxidative stress [11,12]. Moreover, overactivation of the mammalian target of rapamycin complex 1 (mTORC1) signaling pathway, potentially triggered by milk intake, has been linked to increased risks of obesity, type 2 diabetes, cancer, and neurodegenerative diseases [10,13,14]. The health effects of dairy may further vary depending on compositional differences influenced by factors such as animal feeding practices and processing methods. These mixed findings underscore the need for further research to clarify the biological mechanisms by which dairy intake may influence chronic disease risk.

Although interest in the link between diet and telomere biology is growing, few studies have specifically investigated the relationship between dairy consumption and TL. This gap is especially noticeable in Asian populations, where dietary habits vary from Western contexts and where hypertension prevalence is rising. Emerging evidence suggests a complex interaction among diet, TL, and hypertension risk. For example, higher intake of vegetables has been associated with longer TL and lower risk of hypertension [15]. Conversely, shortened telomeres have been linked to increased risks of hypertension and coronary artery disease, reinforcing the potential value of TL as a biomarker for cardiovascular health [16]. Despite these insights, the role of dairy consumption in modulating telomere dynamics remains poorly understood. Given the nutritional and metabolic complexity of dairy products, along with the growing burden of hypertension among aging populations in Asia, clarifying this association may offer valuable implications for dietary strategies aimed at promoting healthy aging. Therefore, the present study examines the association between dairy consumption and TL in normotensive and hypertensive Taiwanese adults, using buccal cell samples to evaluate potential differences in telomere dynamics across blood pressure groups.

## 2. Methods

### 2.1. Study population

**Fig. 1:**
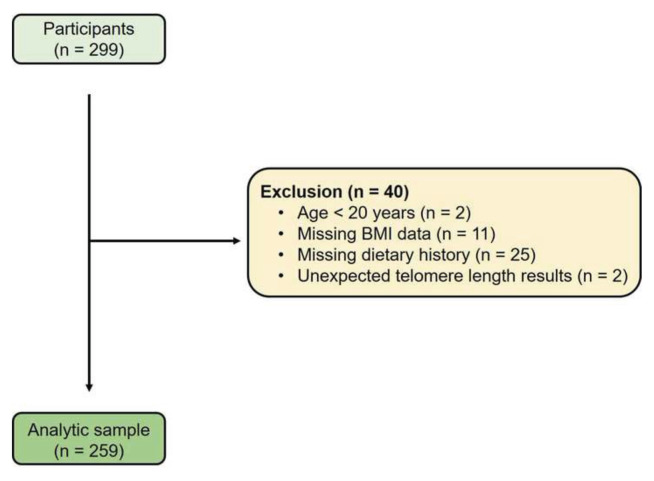
Flowchart of participant enrollment. Of the 299 individuals who completed the survey, 40 were excluded due to age under 20 (n = 2), abnormally long telomere length (n = 2), or missing covariate data (n = 36). The final sample included 259 participants with complete relative telomere length (RTL) and dietary information.

This cross-sectional health survey was conducted during the 2024 Healthy Ageing Tech Show in Taipei, Taiwan, from August 2 to August 4, 2024. Participants were voluntarily recruited on-site through convenience sampling. The aim of the study was to investigate the association between lifestyle factors, dietary habits, and RTL in the Taiwanese population. A total of 299 individuals initially participated in the survey. After data screening, 40 respondents were excluded: 2 individuals were under 20 years of age, 2 exhibited abnormally long telomeres, and 36 had incomplete covariates. As a result, 259 eligible participants with complete RTL and dietary information were included in the final analysis (Fig. 1).

### 2.2. Assessment of dietary intake

Dietary intake was evaluated using a self-reported questionnaire covering participants’ food consumption over the past four weeks. This questionnaire included approximately 10 food categories, encompassing protein sources, carbohydrates, vegetables, and fruits. For each food item, participants indicated their intake frequency, which ranged from “less than once in the past four weeks” to “four or more times per day,” with portion sizes based on standardized references.

To assess dairy consumption, participants were asked about both the frequency and types of dairy products they consumed over the past month. Frequency was classified into three categories: never, occasionally (a few times per week), and frequently (almost daily). The selection of dairy product categories was informed by existing literature on their potential impact on blood pressure and hypertension. These categories included total dairy intake (sum of milk, buttermilk, and yogurt intakes), as well as specific dairy products such as milk, buttermilk, yogurt, and different fat content variations (full-fat, low-fat, and fat-free).

Multiple imputation was utilized to minimize potential bias arising from incomplete data to address the missing data concerning dairy product types. These values were estimated using regression-based imputation models for variables with missing values-such as milk, buttermilk, yogurt, and various fat content variations. The imputation process was conducted five times to create multiple complete datasets. Final analyses integrated the estimates and standard errors from these datasets to account for the uncertainty introduced by the imputation process. This method enhances the robustness and accuracy of statistical inferences.

### 2.3. Telomere length measurement

To collect buccal cells, scrape the inside of the mouth 10 times with a buccal brush. Buccal cells were stored in a test tube at 4°C for up to one week. TL was analyzed by Relative Human Telomere Length Quantification qPCR Assay Kit (ELK Biotechnology, Denver, CO, USA) according to the Manufacturer’s instruction. This assay amplifies a 78-base pair (bp) telomere sequence located on human chromosome 11 and compares it to reference genomic DNA. The amplification is monitored using SYBR Green dye, with multiple cycles detecting TL. The results are expressed as the T/S ratio that is calculated by dividing the telomere quantity (T) by the reference quantity of a single-copy gene (S) to normalize the data. The T/S ratio represents the relative TL (RTL) of the sample. To ensure data standardization, T/S ratio data were log-converted.

### 2.4. Covariates assessment

The study considered various potential covariates, including sociodemographic variables such as age, sex, and educational attainment (categorized as education below high school, high school diploma or equivalent, and above college graduate). Lifestyle variables included smoking status (never, former, current), alcohol consumption (never, former, current), physical activity, and body mass index (BMI). BMI was calculated by dividing weight (kg) by height (m^2^) and categories were defined as underweight (BMI<18.5 kg/m^2^), normal weight (BMI = 18.5–23.9 kg/m^2^), overweight (BMI = 24.0–26.9 kg/m^2^), and obese (BMI≥27 kg/ m^2^). Physical activity was classified into four levels: no aerobic activity, low activity (predominantly sitting during the day and infrequent activity), moderate activity (standing or walking all the time during the day without frequent extraction of items, or carrying lightweight items or frequent mountain climbing), and high activity (having to work at high loads or carrying heavy objects). Past medical history including hypertension, diabetes mellitus, and heart disease were identified through participants’ self-reports.

### 2.5. Statistical analysis

Data normality was assessed using histograms, and variables were log-transformed when necessary. Continuous variables were presented as mean ± standard error, while categorical variables were expressed as counts and proportions. Demographic and lifestyle characteristics of the participants were categorized to display frequency distributions where appropriate. Mean RTL across different demographic and lifestyle categories was compared using analysis of variance (ANOVA) or independent t-tests.

To examine potential subgroup differences, stratified analyses were performed based on participants’ hypertension status. Linear regression was conducted to evaluate the association between RTL and each covariate, including dairy product consumption. To further investigate the relationship between RTL and dairy product consumption, multivariable models were constructed to assess the effect of a one-standard deviation (SD) increase in dairy intake. We developed three linear regression models to evaluate the relationship between dairy product intake and RTL in hypertension. The first model provided a crude estimate of this association, while the second model was further adjusted for general demographic characteristics, including age, sex education level, and BMI. The third model additionally controlled for lifestyle factors, including smoking, alcohol consumption, and physical activity. All statistical tests conducted were two-sided, with statistical significance defined as p < 0.05. Statistical analyses were performed using SPSS software version 29.0 (IBM Corp., Armonk, NY, USA).

## 3. Results

### 3.1. Characteristics of the study population and factors associated with RTL

The study included a total of 259 participants, comprising 80 males (30.9 %) and 179 females (69.1 %), with a mean age of 58.99 ± 0.94 years. The average RTL, measured as the log-transformed T/S ratio, was 2.07 ± 0.014. The study population exhibited variability in RTL, demographic characteristics, lifestyle factors, and the presence of chronic diseases. A detailed summary of participant characteristics is shown in Table 1.

Across different age groups, participants aged 20–40 years had an average T/S ratio of 2.16 ± 0.041, those aged 41–65 years had a T/S ratio of 2.09 ± 0.021, and individuals over 65 years had a T/S ratio of 2.02 ± 0.020. The association between age and RTL was statistically significant ( *p* < 0.01), with RTL progressively decreasing as age increased. However, no significant differences in mean RTL were observed based on sex, education level, body mass index (BMI), or lifestyle factors such as smoking status, alcohol consumption, or regular physical activity. Notably, RTL was significantly associated with a medical history of hypertension ( *p* < 0.05). Participants with hypertension had a shorter mean RTL compared to those without hypertension (2.03 ± 0.025 vs. 2.08 ± 0.016).

### 3.2. Dietary patterns and food group correlations in the study population

Based on questionnaire responses, nearly 90 % of participants reported consuming dairy products at least once per week, while only 10.4 % reported never consuming them. Rice and noodles were staple foods, with 79.9 % of participants consuming more than one serving per day, although only 1.5 % reported consuming more than four servings daily. Protein intake showed inter-individual variation: 58.3 % of participants consumed 1–2 servings of legumes or soy products per day, and 39.4 % reported similar intake levels for seafood. In contrast, fruit consumption was relatively low, as 74.9 % consumed fewer than two servings per day, and only 20.4 % met the recommended intake of 2–3 servings daily. However, vegetable intake was notably higher, with over 80 % of participants consuming at least three servings per day (Supplemental Table S1) (https://www.biomedicinej.com/cgi/editor.cgi?article=1692&window=additional_files&context=biomedicine).

**Fig. 2:**
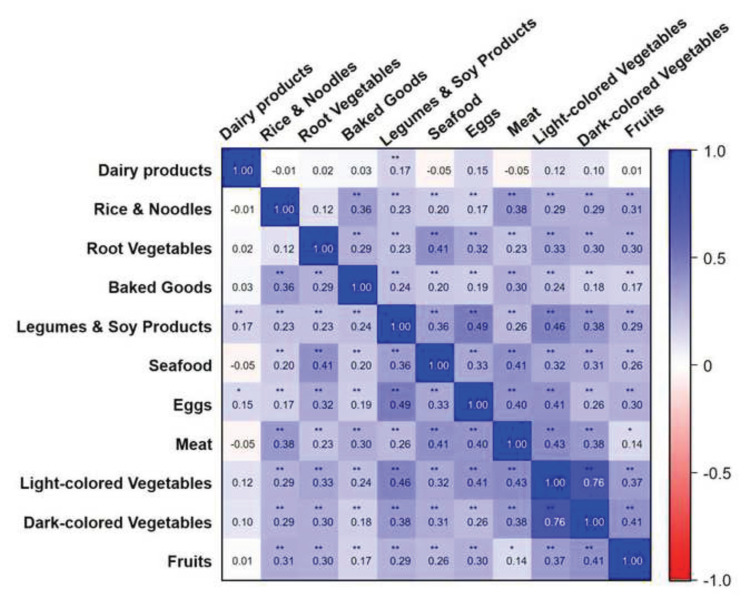
Pearson correlation matrix between each type of food group. Each cell displays the Pearson correlation coefficient (r) between pairs of food groups, with color shading indicating the strength and direction of the correlation. Dark blue represents a strong positive correlation, whereas dark red represents a strong negative correlation. *Significance levels: *p < 0.05; **p < 0.01.

To examine dietary behavior patterns more systematically, we conducted Spearman correlation analyses among food group intakes (Fig. 2).Asignificant positive correlation was observed between vegetable (both light- and dark-colored) and fruit consumption (*r* > 0.3, *p* < 0.01), indicating a trend toward healthy dietary habits. Similarly, legume and soy product intake showed moderate correlations with both fruit (*r*= 0.29, *p* < 0.01) and vegetable intake (*r*= 0.38–0.46, *p* < 0.01), suggesting that plant-based proteins are often consumed alongside nutrient-rich plant foods. Similarly, seafood intake was positively associated with vegetable consumption (*r* > 0.3, *p* < 0.01), supporting the clustering of health-conscious dietary components. In contrast, meat consumption demonstrated a moderate correlation with egg intake (*r* = 0.40) and baked goods (*r* = 0.30), potentially reflecting a pattern of higher energy-dense, protein-rich dietary profiles. However, dairy product intake showed only very weak to weak correlations with other food categories (*r* < 0.2), suggesting that dairy consumption in this population may occur independently of broader dietary quality patterns.

### 3.3. Differential associations of age and dairy consumption with RTL in normotensive and hypertensive individuals

To explore the impact of multiple factors on RTL, we performed a multiple regression analysis. When stratified the analysis by blood pressure status, age was significantly associated with shorter RTL in the normotensive group (β = −0.003, 95 % CI: −0.005, −0.001, *p* < 0.01), suggesting that RTL declines with increasing age. However, this association was not statistically significant in the hypertensive group (β = −0.004, 95 % CI: −0.009, 0.002, *p* = 0.195). The difference in the age-telomere length association between normotensive and hypertensive individuals was not statistically significant (z N vs H = 0.32, *p* = 0.655). Furthermore, other demographic and lifestyle factors did not exhibit significant associations with RTL ( *p* > 0.05) (Table 2).

The estimated associations between nutritional status and relative RTL are presented in Table 2. Our analysis revealed that carbohydrate intake, protein intake, vegetable intake, and fruit intake were not significantly associated with RTL in either group. Interestingly, the frequency of dairy product consumption was significantly associated with shorter RTL, but only in the normotensive group (β = −0.079, 95 % CI: −0.146, −0.019, *p* < 0.05), while no significant association was observed in the hypertensive group (β = −0.045, 95 % CI: −0.125, 0.041, *p* = 0.301). The difference in the association between dairy consumption and RTL between the two groups was statistically significant (z N vs. H = −2.060, *p* = 0.039).

### 3.4. Inverse association between dairy product consumption and RTL in normotensive individuals

**Fig. 3:**
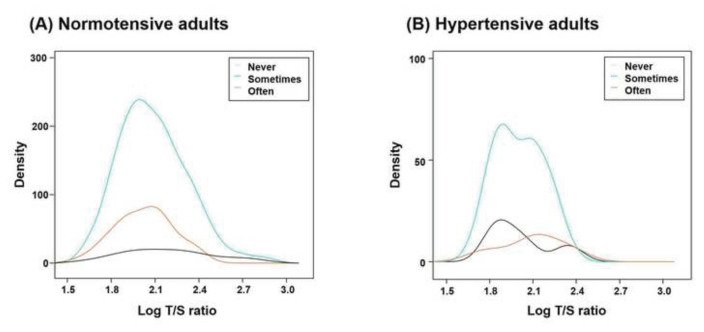
Kernel density plots depicting the marginal distribution of log-transformed relative telomere length (RTL) based on dairy consumption frequency. The density curves illustrate: (A–B) the Log T/S ratio for different frequencies of dairy consumption (Never, Sometimes, Often) among (A) normotensive adults and (B) hypertensive adults.

Kernel density plots were used to visualize the distribution of RTL according to dairy consumption frequency (Fig. 3). In the normotensive group, those who reported consuming dairy sometimes or often exhibited a rightward shift in RTL distribution, indicating shorter telomere length compared to those who never consumed dairy (Fig. 3A). In contrast, this pattern was less evident in hypertensive adults, with minimal differences observed across the dairy consumption groups (Fig. 3B).

Finally, we examined the association between different types of dairy product consumption and RTL s across different blood pressure groups (Table 3). Among normotensive participants, milk consumption frequency was significantly associated with RTL ( *p* < 0.05), with those consuming milk often exhibiting shorter telomeres compared to those who never consumed milk. In addition, RTL varied significantly by the fat content of dairy products ( *p* < 0.05), with individuals consuming low-fat or fat-free dairy showing shorter RTL than those consuming full-fat dairy. In contrast, no significant associations were observed between dairy consumption and RTL among hypertensive participants.

After adjusting for demographics and lifestyle factors, a significant inverse association was observed between the frequency of dairy product consumption and RTL among normotensive participants (β = −0.082, *p* < 0.01) (Table 4). Similar results were found for dairy fat content, where higher intake of low-fat or fat-free dairy was associated with shorter RTL (β = −0.106, *p* < 0.01). These associations remained robust across all adjustment models. Notably, no significant associations were found between dairy variables and RTL in the hypertensive group. This pattern suggests that the impact of dairy intake on cellular aging may be more pronounced in the earlier stages of cardiometabolic health, highlighting the potential importance of early dietary interventions before hypertension develops.

## 4. Discussion

The health effects of milk consumption remain contested. While milk is a rich source of essential nutrients such as calcium, protein, and vitamins, it also contains bioactive compounds that may influence disease risk. Evidence suggests milk intake is beneficial in certain contexts, such as improving infant birth weight during pregnancy, supporting bone health during childhood, and reducing frailty in older adults [17]. However, long-term milk consumption has also been associated with increased risk of obesity, insulin resistance, neurodegenerative disorders, and certain cancers [18,19]. A prospective 30-year cohort study further reported that higher milk consumption during midlife was associated with an elevated risk of Parkinson’s disease, independent of dietary calcium intake [20]. These contradictory findings underscore the need to understand the contextual and mechanistic factors mediating the health effects of dairy products.

TL is widely recognized as a robust biomarker of biological aging, with progressive shortening linked to cellular senescence, genomic instability, and chronic disease risk [21]. In this study, we observed a significant inverse association between dairy consumption frequency and RTL, but only among normotensive individuals. This pattern was not evident in participants with hypertension, suggesting a possible interaction between blood pressure status and dietary effects on telomere biology. These findings align with prior studies associating high-fat dairy intake with accelerated telomere attrition, elevated levels of oxidative and inflammatory biomarkers in serum, and increased allcause mortality [22–24].

Oxidative stress and chronic low-grade inflammation are key drivers of both telomere shortening and the pathogenesis of age-related conditions such as metabolic syndrome [25]. The absence of an observed association between dairy intake and telomere length in hypertensive individuals may reflect a physiological ceiling effect. That is, elevated baseline levels of oxidative and inflammatory activity in this population may limit the detectability of any additional impact from dairy consumption on telomere attrition. In other words, when oxidative stress and inflammation are already high, further dietary exposures may have negligible or undetectable effects on telomere dynamics. This interpretation is supported by the lack of significant RTL differences across dairy intake categories in the hypertensive group, despite a strong inverse association observed in normotensive participants.

We also observed an inverse association between low-fat dairy consumption and telomere length. One mechanistic explanation involves galactose, a component of lactose. Fat-reduced milk has a higher concentration of lactose per volume, which is hydrolyzed into glucose and galactose in humans [26]. Animal models have shown that chronic exposure to d-galactose induces oxidative stress, inflammation, neurodegeneration, and cellular aging [27,28]. In humans, galactose has been implicated in non-enzymatic glycation and the accumulation of advanced glycation end-products (AGEs), contributing to tissue aging [29]. Additionally, high intake of saturated and trans fats which present in some dairy products has been linked to shorter telomeres, cognitive decline, and systemic inflammation [30–32]. Taken together, these findings suggest that milk intake, particularly in its low-fat form, may accelerate biological aging through pathways involving galactose metabolism and fat-induced oxidative stress.

The context-dependent nature of dairy’s health effects is further underscored by previous research showing antihypertensive properties of milk-derived bioactive peptides. Clinical and experimental studies have demonstrated that milk-derived bioactive peptides, particularly the tripeptides isoleucine-proline-proline (IPP), and valine-prolineproline (VPP) may act as angiotensin-converting enzyme (ACE) inhibitors, reducing systolic and diastolic blood pressure, particularly in hypertensive individuals [33,34]. However, the broader literature remains inconclusive, with meta-analyses reporting inconsistent associations between milk consumption and cardiovascular outcomes [35]. These inconsistencies may be due to differences in dairy composition, which can be influenced by animal feed and processing methods [36].

From a public health perspective, our findings highlight the need for more individualized dietary recommendations. While dairy intake may offer benefits for blood pressure management, its potential role in accelerating cellular aging among normotensive individuals requires careful consideration. Future guidelines should account for underlying metabolic status and chronic disease risk when evaluating the long-term health implications of dairy consumption. One possible mechanism underlying the association between diet and cellular aging is oxidative stress and inflammation. Antioxidants and anti-inflammatory dietary components, such as those found in fruits, vegetables, whole grains, and soya, have been shown to reduce inflammatory biomarkers and may help preserve telomere length during aging [37–39]. Understanding these biological pathways is essential for developing more targeted dietary interventions to mitigate age-related health risks [40].

Despite revealing an inverse association between dairy consumption and telomere length in normotensive individuals, this study has several limitations. First, dietary intake was based on a one-month recall using self-reported questionnaires. Short-term dietary recall which may be subject to recall bias and may not accurately reflect participants’ long-term dietary habits. Besides, the cross-sectional design precludes causal inference, making it impossible to determine the directionality of the observed associations, longitudinal and interventional studies are needed to clarify directionality. Thirdly, although buccal cell sampling offers a minimally invasive approach for large-scale studies, they may contain mixed cell types, including epithelial and leukocyte populations, potentially introducing measurement variability. Future studies should consider cellular composition quantification or compare telomere lengths across multiple tissues. Fourthly, the study did not include biochemical markers of oxidative stress or inflammation, which limits our ability to explore the mechanistic underpinnings of telomere attrition associated with dairy intake. Given that oxidative stress and chronic low-grade inflammation are key mediators of both hypertension and TL shortening, incorporating biomarkers such as C-reactive protein, IL-6, and 8-OHdG in future studies would provide deeper insights. Finally, participants were recruited via convenience sampling at the 2024 Healthy Ageing Tech Show in Taipei, which may introduce selection bias due to the likelihood of attracting health-conscious individuals. Future studies should employ random sampling across diverse settings to enhance population representativeness.

## 5. Conclusions

This study provides novel evidence of an inverse association between dairy product consumption and RTL in normotensive adults, but not in those with hypertension. The findings suggest that the biological impact of dairy intake on cellular aging may vary by blood pressure status, potentially due to underlying metabolic or inflammatory differences. These results highlight the importance of considering individual health profiles when developing dietary recommendations. Public health strategies aiming to promote healthy aging should account for the nuanced role of dairy products, particularly low-fat and fat-free varieties, in telomere dynamics.

## Supplementary Information



## Figures and Tables

**Table 1:** Descriptive characteristics of participants and RTL, stratified by demographic and health variables.

Characteristics	All participants (n = 259)		

	n (%) or mean ± SE	RTL, mean ± SE	*p*
Age (years), mean ± SE	58.99 ± 0.94	2.073 ± 0.014	<0.01
Age category, n (%)			
20–40	36 (13.9)	2.157 ± 0.041	
41–65	118 (45.6)	2.091 ± 0.021	
>65	105 (40.5)	2.023 ± 0.020	
Sex, n (%)			0.219
Male	80 (30.9)	2.056 ± 0.024	
Female	179 (69.1)	2.080 ± 0.017	
Education level, n (%)			0.911
Low	18 (7.0)	2.064 ± 0.053	
Middle	49 (18.9)	2.062 ± 0.037	
High	192 (74.1)	2.076 ± 0.016	
Body mass index (kg/m^2^), mean ± SE	23.27 ± 0.20	2.073 ± 0.014	0.453
Body mass index category			
Underweight (<18.5)	15 (5.8)	2.14 ± 0.065	
Normal weight (18.5–24.9)	178 (68.7)	2.06 ± 0.016	
Overweight (25.0–29.9)	60 (23.2)	2.09 ± 0.033	
Obesity (30.0), n (%)	6 (2.3)	1.99 ± 0.061	
Smoking status, n (%)			0.627
Never	245 (94.6)	2.076 ± 0.015	
Former	9 (3.5)	2.010 ± 0.077	
Current	5 (1.9)	2.026 ± 0.096	
Alcohol consumption, n (%)			0.224
Never	229 (88.4)	2.069 ± 0.015	
Current	30 (11.6)	2.102 ± 0.048	
Regular physical activity, n (%)			0.393
No aerobic activity	59 (22.8)	2.057 ± 0.218	
Low activity	88 (34.0)	2.085 ± 0.243	
Moderate activity	78 (30.1)	2.086 ± 0.194	
High activity	34 (13.1)	2.087 ± 0.271	
Chronic disease			
Hypertension, n (%)			<0.05
Yes	52 (20.1)	2.025 ± 0.025	
No	207 (79.1)	2.085 ± 0.016	
Diabetes mellitus, n (%)			0.080
Yes	26 (10.0)	2.013 ± 0.037	
No	233 (90.0)	2.079 ± 0.015	
Heart disease, n (%)			0.282
Yes	17 (6.6)	2.104 ± 0.050	
No	242 (93.4)	2.070 ± 0.015	

Abbreviations: RTL = log-transformed relative telomere length; SE = standard error.

**Table 2:** Linear regression of demographic, lifestyle, and dietary factors associated with RTL, stratified by hypertension status.

	Normotensive group (n = 207)		Hypertensive group (n = 52)	
	
	β (95 % CI)	*p*	β (95 % CI)	*p*
Age	−0.003 (−0.005, −0.001)	**0.007** **	−0.004 (−0.009, 0.002)	0.195
Sex	0.041 (−0.030, 0.112)	0.255	−0.049 (−0.156, 0.058)	0.364
Education level	0.002 (−0.057, 0.061)	0.936	0.000 (−0.072, 0.071)	0.992
BMI	−0.002 (−0.013, 0.009)	0.736	0.003 (−0.013, 0.020)	0.696
Smoking status	−0.054 (−0.180, 0.073)	0.406	0.000 (−0.101, 0.102)	0.996
Alcohol consumption	0.021 (−0.033, 0.074)	0.451	0.020 (−0.048, 0.088)	0.563
Regular physical activity	0.008 (−0.026, 0.041)	0.654	0.010 (−0.045, 0.065)	0.717
Dairy production	−0.079 (−0.146, −0.019)	**0.01** *	−0.045 (−0.125, 0.041)	0.301
Carbohydrate intake	0.037 (−0.022, 0.097)	0.220	0.028 (−0.057, 0.114)	0.507
Rice & Noodles	0.016 (−0.026, 0.059)	0.447	0.018 (−0.039, 0.076)	0.518
Root vegetables	−0.037 (−0.094, 0.021)	0.212	−0.062 (−0.135, 0.011)	0.094
Baked goods	−0.001 (−0.057, 0.055)	0.967	−0.029 (−0.101, 0.042)	0.413
Protein intake	−0.003 (−0.087, 0.081)	0.942	−0.026 (−0.127, 0.076)	0.614
Legumes & Soy products	−0.02 (−0.063, 0.023)	0.364	−0.019 (−0.073, 0.036)	0.496
Seafood	−0.02 (−0.075, 0.035)	0.474	−0.047 (−0.121, 0.026)	0.203
Eggs	−0.009 (−0.061, 0.044)	0.740	−0.019 (−0.085, 0.047)	0.569
Meat	0.01 (−0.036, 0.056)	0.673	−0.005 (−0.067, 0.056)	0.870
Vegetable intake	−0.029 (−0.071, 0.013)	0.180	0.03 (−0.025, 0.085)	0.275
Light-colored vegetables	−0.025 (−0.065, 0.016)	0.233	0.016 (−0.041, 0.073)	0.585
Dark-colored vegetables	−0.016 (−0.055, 0.023)	0.411	0 (−0.056, 0.056)	0.995
Fruits intake	0.006 (−0.071, 0.083)	0.876	0.092 (−0.015, 0.199)	0.090

Abbreviations: RTL = log-transformed relative telomere length; CI = confidence interval; BMI = body mass index.

* Significance levels:

* *p* < 0.05;

** *p* < 0.01.

**Table 3:** Comparison of RTL across levels of dairy product consumption in normotensive and hypertensive participants.

	Normotensive group			Hypertensive group		
	
	n (%)	RTL	*p*	n (%)	RTL	*p*
Frequency of milk^a^			**0.043** *			0.498
Never	18 (8.7)	2.191 ± 0.072		9 (17.3)	2.018 ± 0.069	
Sometimes	144 (69.6)	2.089 ± 0.020		35 (67.3)	2.011 ± 0.028	
Often	45 (21.7)	2.028 ± 0.029		8 (15.4)	2.096 ± 0.077	
Total dairy			0.127			0.873
No	18 (14.2)	2.191 ± 0.072		9 (27.3)	2.018 ± 0.069	
Yes	109 (85.8)	2.092 ± 0.023		24 (72.7)	2.030 ± 0.037	
Milk			0.927			0.115
No	51 (40.2)	2.109 ± 0.035		15 (45.5)	1.971 ± 0.048	
Yes	76 (59.8)	2.105 ± 0.030		18 (54.5)	2.073 ± 0.041	
Buttermilk			0.442			0.383
No	92 (72.4)	2.117 ± 0.028		26 (78.8)	2.041 ± 0.034	
Yes	35 (27.6)	2.078 ± 0.038		7 (21.2)	1.971 ± 0.086	
Yogurt			0.328			0.821
No	99 (78)	2.118 ± 0.026		28 (84.8)	2.023 ± 0.035	
Yes	28 (22)	2.065 ± 0.042		5 (15.2)	2.044 ± 0.084	
Content of fat			**0.021** *			0.933
None	19 (15.1)	2.180 ± 0.069		9 (31)	2.018 ± 0.069	
Full-fat	69 (54.8)	2.118 ± 0.028		18 (62.1)	2.036 ± 0.047	
Low-fat or fat-free	38 (30.2)	2.007 ± 0.037		2 (6.9)	2.075 ± 0.165	

Note. RTL (log-transformed relative telomere length) is presented as mean ± SE; SE = standard error.

* Significance levels:

* *p* < 0.05;

** *p* < 0.01.

a The frequency of milk consumption was categorized into three levels: (1) Never, which included participants who reported no milk consumption; (2) Sometimes, defined as adults who consumed milk at least once per week but less than daily; and (3) Often, referring to individuals who consumed milk once per day.

**Table 4:** Multivariable regression models examining the association between dairy intake and RTL.

	Model^b^	Normotensive group		Hypertensive group	
	
		β (95 % CI)	*p*	β (95 % CI)	*p*
Frequency of dairy production^a^	1	−0.075 (−0.135, −0.015)	0.014*	0.037 (−0.052, 0.127)	0.406
	2	−0.081 (−0.141, −0.020)	0.009**	0.042 (−0.053, 0.136)	0.381
	3	−0.082 (−0.143, −0.022)	0.008**	0.043 (−0.057, 0.142)	0.391
Total dairy production	1	−0.099 (−0.226, 0.029)	0.127	0.012 (−0.138, 0.161)	0.873
	2	−0.125 (−0.258, 0.008)	0.065	0.006 (−0.154, 0.165)	0.944
	3	−0.133 (−0.266, −0.001)	0.049*	0.005 (−0.168, 0.179)	0.95
Milk	1	−0.004 (−0.096, 0.087)	0.927	0.102 (−0.026, 0.230)	0.115
	2	−0.025 (−0.116, 0.067)	0.593	0.095 (−0.041, 0.230)	0.164
	3	−0.021 (−0.113, 0.072)	0.659	0.103 (−0.043, 0.248)	0.158
Buttermilk	1	−0.039 (−0.139, 0.061)	0.442	−0.070 (−0.230, 0.091)	0.383
	2	−0.037 (−0.137, 0.063)	0.467	−0.051 (−0.221, 0.120)	0.547
	3	−0.028 (−0.128, 0.072)	0.579	−0.061 (−0.245, 0.123)	0.501
Yogurt	1	−0.053 (−0.161, 0.054)	0.328	0.021 (−0.165, 0.206)	0.821
	2	−0.053 (−0.161, 0.054)	0.329	0.063 (−0.140, 0.266)	0.53
	3	−0.048 (−0.155, 0.060)	0.383	0.058 (−0.166, 0.283)	0.597
Content of fat	1	−0.091 (−0.156, −0.026)	0.006**	0.024 (−0.111, 0.159)	0.72
	2	−0.098 (−0.162, −0.034)	0.003**	−0.019 (−0.163, 0.125)	0.787
	3	−0.106 (−0.170, −0.042)	0.001**	−0.010 (−0.167, 0.148)	0.901

Abbreviations: RTL = log-transformed relative telomere length; CI = confidence interval.

* Significance levels:

* *p* < 0.05;

** *p* < 0.01.

a The frequency of milk consumption was categorized into three levels: (1) Never, which included participants who reported no milk consumption; (2) Sometimes, defined as adults who consumed milk at least once per week but less than daily; and (3) Often, referring to individuals who consumed milk once per day.

b The models were adjusted for the following covariates: Model 1 was the crude (not adjusted) model. Model 2 was adjusted for age, sex, body mass index (BMI), and educational attainment. Model 3 included additional adjustments for alcohol consumption, smoking status, and physical activity, in addition to the covariates in Model 2.
