# Supplementary material for: Increased dairy product consumption is associated with shorter telomere length in buccal cells among normotensive adults
Source: Biomedicine (Taipei). 2026 Mar 1;16(1):1–11. doi: 10.37796/2211-8039.1692 (PMC12962758; doi:10.37796/2211-8039.1692)
Supplement: Supplementary file 1 [file bmed-16-01-001-s001.docx]

**Supplementary Materials**

**Supplemental Table 1.** Frequency of food group consumption according to questionnaire responses

*Supplemental Table 1. Frequency of food group consumption according to questionnaire responses*

| Food groups | Frequency | Total | Hypertension, n (%) | |
| --- | --- | --- | --- | --- |
|  |  |  | No | Yes |
| Dairy products ^a^ | Never | 27 | 18 (8.7) | 9 (17.2) |
|  | Sometimes | 179 | 144 (69.6) | 35 (67.3) |
|  | Often | 53 | 45 (21.7) | 8 (15.4) |
| Rice & Noodles | Less than one serving per day | 52 | 41 (19.80) | 11 (21.20) |
|  | 1–2 servings per day | 138 | 113 (54.60) | 25 (48.10) |
|  | 2–3 servings per day | 60 | 48 (23.20) | 12 (23.10) |
|  | 3–4 servings per day | 5 | 2 (1.00) | 3 (5.80) |
|  | 4 or more servings per day | 4 | 3 (1.40) | 1 (1.90) |
| Root Vegetables | Less than one serving per day | 155 | 121 (58.50) | 34 (65.40) |
|  | 1–2 servings per day | 98 | 81 (39.10) | 17 (32.70) |
|  | 2–3 servings per day | 4 | 4 (1.90) | 0 (0.00) |
|  | 3–4 servings per day | 1 | 1 (0.50) | 0 (0.00) |
|  | 4 or more servings per day | 1 | 0 (0.00) | 1 (1.90) |
| Baked Goods | Less than one serving per day | 166 | 132 (63.80) | 34 (65.40) |
|  | 1–2 servings per day | 85 | 69 (33.30) | 16 (30.80) |
|  | 2–3 servings per day | 5 | 4 (1.90) | 1 (1.90) |
|  | 3–4 servings per day | 2 | 2 (1.00) | 0 (0.00) |
|  | 4 or more servings per day | 1 | 0 (0.00) | 1 (1.90) |
| Legumes & Soy Products | Less than one serving per day | 71 | 50 (24.20) | 21 (40.40) |
|  | 1–2 servings per day | 151 | 125 (60.40) | 26 (50.00) |
|  | 2–3 servings per day | 26 | 25 (12.10) | 1 (1.90) |
|  | 3–4 servings per day | 6 | 4 (1.90) | 2 (3.80) |
|  | 4 or more servings per day | 5 | 3 (1.40) | 2 (3.80) |
| Seafood | Less than one serving per day | 148 | 122 (58.90) | 26 (50.00) |
|  | 1–2 servings per day | 102 | 77 (37.20) | 25 (48.10) |
|  | 2–3 servings per day | 7 | 7 (3.40) | 0 (0.00) |
|  | 3–4 servings per day | 1 | 1 (0.50) | 0 (0.00) |
|  | 4 or more servings per day | 1 | 0 (0.00) | 1 (1.90) |
| Eggs | Less than one serving per day | 35 | 18 (8.70) | 17 (32.70) |
|  | 1–2 servings per day | 183 | 153 (73.90) | 30 (57.70) |
|  | 2–3 servings per day | 31 | 28 (13.50) | 3 (5.80) |
|  | 3–4 servings per day | 8 | 7 (3.40) | 1 (1.90) |
|  | 4 or more servings per day | 2 | 1 (0.50) | 1 (1.90) |
| Meat | Less than one serving per day | 62 | 45 (21.70) | 17 (32.70) |
|  | 1–2 servings per day | 146 | 120 (58.00) | 26 (50.00) |
|  | 2–3 servings per day | 44 | 37 (17.90) | 7 (13.50) |
|  | 3–4 servings per day | 6 | 5 (2.40) | 1 (1.90) |
|  | 4 or more servings per day | 1 | 0 (0.00) | 1 (1.90) |
| Light-colored Vegetables | Less than one serving per day | 32 | 21 (10.10) | 11 (21.20) |
|  | 1–2 servings per day | 154 | 129 (62.30) | 25 (48.10) |
|  | 2–3 servings per day | 53 | 41 (19.80) | 12 (23.10) |
|  | 3–4 servings per day | 15 | 12 (5.80) | 3 (5.80) |
|  | 4 or more servings per day | 5 | 4 (1.90) | 1 (1.90) |
| Dark-colored Vegetables | Less than one serving per day | 34 | 24 (11.60) | 10 (19.20) |
|  | 1–2 servings per day | 150 | 125 (60.40) | 25 (48.10) |
|  | 2–3 servings per day | 53 | 41 (19.80) | 12 (23.10) |
|  | 3–4 servings per day | 16 | 12 (5.80) | 4 (7.70) |
|  | 4 or more servings per day | 6 | 5 (2.40) | 1 (1.90) |
| Fruits | Less than one serving per day | 62 | 49 (23.67) | 13 (25.00) |
|  | 1–2 servings per day | 132 | 110 (53.14) | 22 (42.31) |
|  | 2–3 servings per day | 53 | 39 (18.84) | 14 (26.92) |
|  | 3–4 servings per day | 5 | 3 (1.45) | 2 (3.85) |
|  | 4 or more servings per day | 7 | 6 (2.90) | 1 (1.92) |

^a^ The frequency of milk consumption was categorized into three levels: (1) Never, which included participants who reported no milk consumption; (2) Sometimes, defined as adults who consumed milk at least once per week but less than daily; and (3) Often, referring to individuals who consumed milk once per day.
